# Inadequate response to antiplatelet therapy in Sneddon's syndrome. Time to re-evaluate management recommendations?^☆^

**DOI:** 10.1016/j.abd.2024.01.001

**Published:** 2024-04-23

**Authors:** Cristóbal Lecaros, Gabriela Coulon, Francisca Reculé, Alex Castro, Constanza Del Puerto

**Affiliations:** a Department of Dermatology, Faculty of Medicine, Clínica Alemana, Universidad del Desarrollo, Santiago, Chile; b Department of Pathology, Faculty of Medicine, Clínica Alemana, Universidad del Desarrollo, Santiago, Chile

Dear Editor,

A 46-year-old woman presented with asymptomatic violaceous macules on her skin for over 20 years. She had hypertension and had suffered an ischemic stroke at age 38, currently treated with enalapril, aspirin, and rosuvastatin. Despite these drugs, she had a second ischemic stroke at age 46, causing secondary dysarthria, anomic aphasia, and right-sided hemiparesis. She also had a long history of migraines since her youth and had experienced one miscarriage. The physical exam revealed asymptomatic broken reticulated violaceous macules on her arms, trunk, buttocks, and legs (Figure 1, Figure 2).

**Figure 1:**
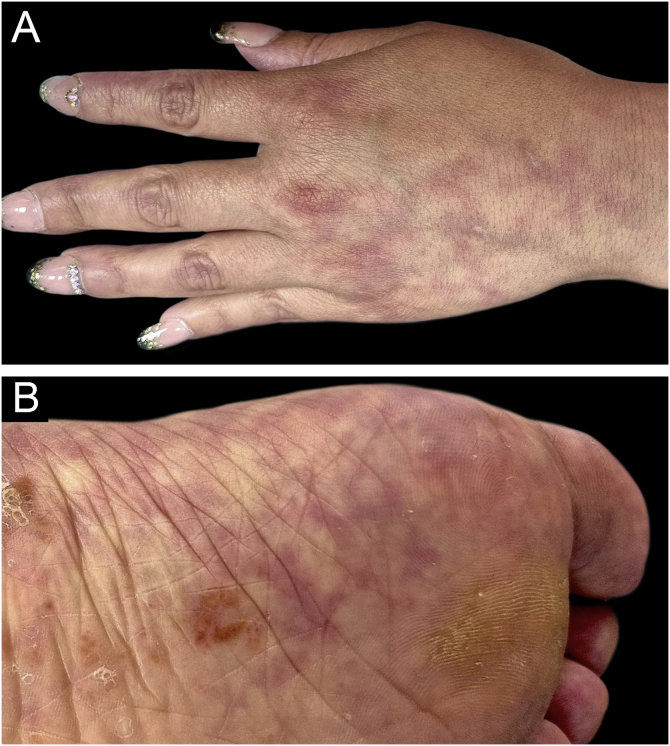
**Livedo racemosa in Sneddon syndrome**. (A‒B) Extended violaceous irregular reticular macules on the hands and trunk.

**Figure 2:**
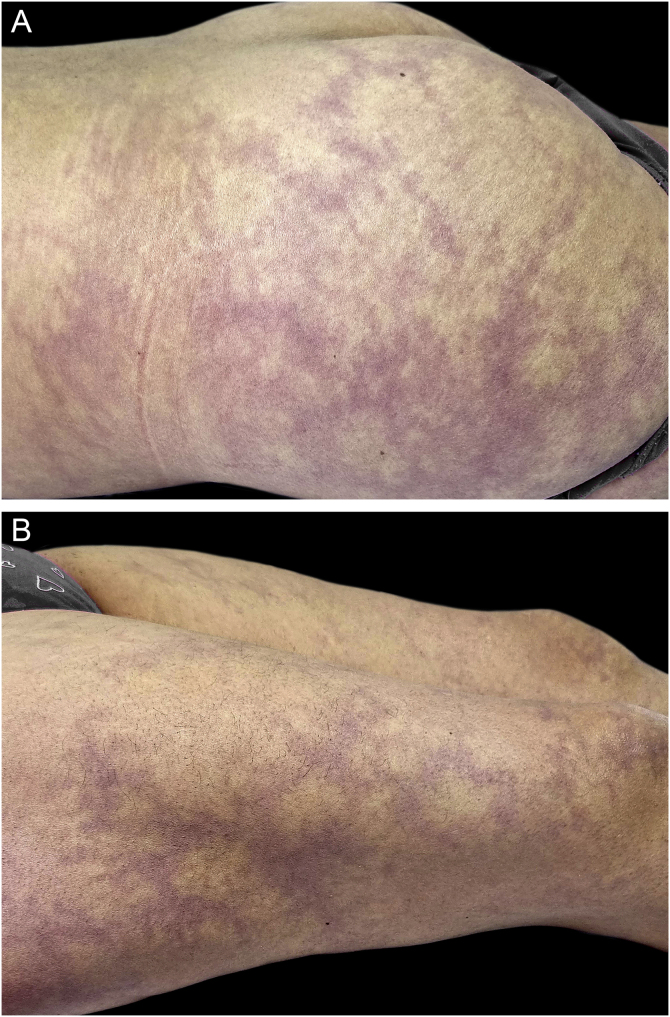
**Livedo racemosa in Sneddon syndrome**. (A‒B) Note the width of the branches (wider than 1 cm) at the buttocks and lower extremities of a patient with Sneddon syndrome and Protein S deficiency.

A wide, deep skin biopsy showed multiple small arteries with thickening of the wall, intimal hyperplasia, stenosis, and lumen obliteration, with secondary signs of re-tunneling and neo-vascularization in the reticular dermis and subcutis (Fig. 3).

**Figure 3:**
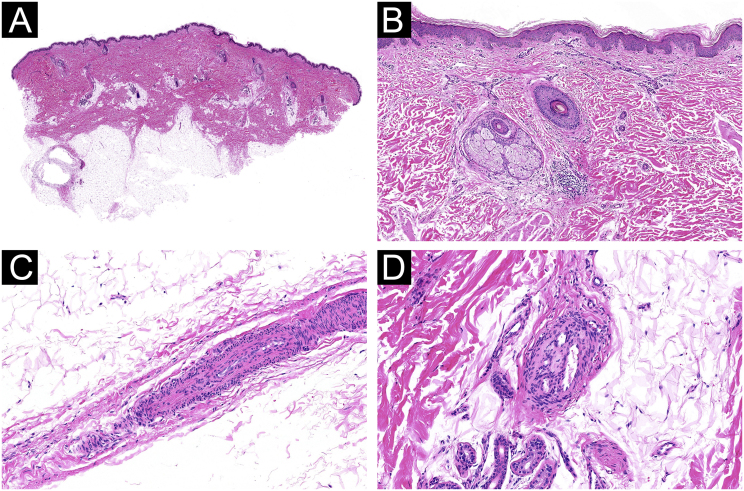
**Histological features of occlusive vasculopathy in Sneddon Syndrome**. (A‒B) Small arteries in the deep dermis and hypodermis with mural thickening and intimal hyperplasia (Hematoxylin & eosin, 4× and 10×). (C) Stenosis and obliteration of the lumen of a vessel (Hematoxylin & eosin, 40×). (D) Signs of re-tunneling and neovascularization without vasculitis (Hematoxylin & eosin, 40×).

Cerebral magnetic resonance angiography revealed infarcts in the left frontal insular, right parietal and right posterior inferior cerebellar areas and parenchymal sequelae in the territory irrigated by the right and left medial cerebral artery. Echocardiogram and 24-h Holter electrocardiography were normal. Prothrombin time was 65%, INR 1.38, ANA 1/80 speckled pattern. Anti-DNA, anti-ENA, ANCA and Antiphospholipid (APL) antibodies were negative. Thrombophilia testing revealed Protein S deficiency. A Sneddon syndrome (SS) APL (-) with Protein S deficiency was diagnosed, and rivaroxaban was initiated.

SS is an underdiagnosed neurocutaneous vasculopathy comprising small and medium-sized arteries, with an estimated incidence of 4 per million per year with a median age of 40 years.^1^ Its etiology is poorly understood, although autoimmune and thrombotic mechanisms have been proposed. An autosomal recessive condition of SS with CERC1 mutations (encoding adenosine deaminase 2) has been described.^2^

SS is characterized by central nervous system manifestations (headaches, multiple strokes, cognitive impairment), persistent livedo racemosa, hypertension, cardiac valvulopathy, and less frequent renal and ocular involvement.^1^, ^3^ SS is classified according to the presence of Anti-Phospholipid (APL) antibodies in two groups: SS_APL-_ and SS_APL+_.^1^

In patients with SS_APL-_ it is important to consider other causes of thrombophilia, as in a recent series, 27% of patients who underwent thrombophilia testing had positive results.^4^ Patients with SS_APL+_ present with thinner branching livedo macules, have a higher frequency of thrombocytopenia, seizures, and chorea.^3^, ^5^ Patients with SS_APL-_ present with a higher number of strokes before diagnosis.^4^

The treatment recommendations differ for patients with SS_APL-_ and SS_APL+_.^3^, ^4^, ^5^, ^6^, ^7^, ^8^ For early stages of SS_APL-_ regular monitoring with brain imaging is recommended, and in case of prodromic symptoms, prescription of low-dose aspirin. After an ischemic event, antiplatelet medications are the first-line treatment.^8^ For SS_APL+_ patients, similar management recommendations are suggested initially, but in the event of arterial thrombosis anticoagulant therapy is recommended.^3^ This difference is based on two retrospective series.^5^, ^6^ Francès et al. followed 46 patients (19 SS_APL+_ and 27 SS_APL-_) for six years.^5^ Authors highlight that among SS_APL+_ patients, those who received anticoagulants had significantly fewer strokes than those on antiplatelet therapy. However, in SS_APL-_ subgroup there were no differences in the number of strokes regardless of therapy.^5^ Bottin et al. followed 53 SS_APL–_ patients for twenty years^6^ and found no significant differences in ischemic events between these two therapies.^6^ Nevertheless, the recently published series by Starmans et al. raises questions regarding these recommendations.^4^ In this retrospective study of 53 patients (14 SS_APL+_ and 39 SS_APL-_) with 21 years of follow-up, some patients were treated with antiplatelet therapy and some with anticoagulation, regardless of APL titers. During follow-up, patients with oral anticoagulation had significantly longer disease-free survival, as the time to first stroke recurrence in this group was twice as long as those receiving antiplatelet therapy (46 vs. 26.5 months, respectively). The authors recommend initiating antiplatelet therapy and switching to oral anticoagulation therapy early after any recurrent ischemic episode.^4^

Our case report emphasizes that an extensive workout is needed in these patients given the higher risk of concomitant thrombophilia. The association between SS and Protein S deficiency has been previously reported.^9^ Also, our case shows that antiplatelet therapy may not be sufficient for disease control in SS_APL-_ patients (Table 1). Given the importance of preventing early onset dementia, direct-acting oral anticoagulants – which have demonstrated similar efficacy to vitamin K antagonists with lower risk of intracranial hemorrhage in patients with ischemic strokes and atrial fibrillation^10^ – should strongly be considered in symptomatic SS_APL-_ patients.

**Table 1:** Reported treatments and outcomes in published series of Sneddon’s syndrome.

Series	Number of patients	Follow up	Therapy^a^	Outcome
Frances et al.^5^	46 patients	6-years	SS_APL+_	0.06 Strokes per year
	19 SS_APL+_		ACO (11)	0.5 Strokes per year
	27 SS_APL-_		Antiplatelet (5)	0.056 Strokes per year
			SS_APL-_	
			ACO (10)	0.08 Strokes per year
			Antiplatelet (18)	
Bottin et al.^6^	56 SS_APL-_ patients	20-years	Antiplatelet (42)	3% stroke recurrence
			ACO (10)	2.7% stroke recurrence
Starmans et al.^4^	31 patients	28-months	ACO	46 months DS
			Antiplatelet	26.5 months DS

ACO, Anticoagulation therapy; DS, Disease Survival; SS_APL-_, Sneddon Syndrome not associated with Antiphospholipid Antibodies; SS_APL+_, Sneddon Syndrome associated with Antiphospholipid Antibodies.

a Number of patients.

## Financial support

None declared.

## Authors’ contributions

Cristóbal Lecaros: The study concept and design; data collection, analysis and interpretation; writing of the manuscript or critical review of important intellectual content; critical review of the literature; final approval of the final version of the manuscript.

Gabriela Coulon: The study concept and design; data collection, analysis and interpretation; writing of the manuscript or critical review of important intellectual content; critical review of the literature; final approval of the final version of the manuscript.

Francisca Reculé: The study concept and design; data collection, analysis and interpretation; writing of the manuscript or critical review of important intellectual content; critical review of the literature; final approval of the final version of the manuscript.

Alex Castro: Intellectual participation in the propaedeutic and/or therapeutic conduct of the studied cases; writing of the manuscript or critical review of important intellectual content; critical review of the literature; final approval of the final version of the manuscript.

Constanza Del Puerto: The study concept and design; data collection, analysis and interpretation; writing of the manuscript or critical review of important intellectual content; intellectual participation in the propaedeutic and/or therapeutic conduct of the studied cases; critical review of the literature; final approval of the final version of the manuscript.

## Conflicts of interest

None declared.
